# Crocus-derived compounds alter the aggregation pathway of Alzheimer’s Disease: associated beta amyloid protein

**DOI:** 10.1038/s41598-020-74770-x

**Published:** 2020-10-23

**Authors:** Nikolaos Stavros Koulakiotis, Pasi Purhonen, Evangelos Gikas, Hans Hebert, Anthony Tsarbopoulos

**Affiliations:** 1 GAIA Research Center, Bioanalytical Department, The Goulandris Natural History Museum, 14562 Kifissia, Greece; 2 School of Engineering Sciences in Chemistry, Biotechnology and Health, Department of Biomedical Engineering and Health Systems, KTH Royal Institute of Technology, S-141 52, Huddinge, Sweden; 3 Department of Pharmacy, National and Kapodistrian University of Athens, 15771 Athens, Greece; 4 Department of Pharmacology, National and Kapodistrian University of Athens Medical School, 75 Mikras Asias Street, 11527 Athens, Greece

**Keywords:** Electron microscopy, Alzheimer's disease, Mass spectrometry

## Abstract

Natural products have played a dominant role in the discovery of lead compounds for the development of drugs aimed at the treatment of human diseases. This electrospray ionization-ion mobility spectrometry-mass spectrometry (ESI-IMS-MS)—based study demonstrates that dietary antioxidants, isolated components from the stigmas of saffron (*Crocus sativus* L.) may be effective in inhibiting Aβ fibrillogenesis, a neuropathological hallmark of Alzheimer’s Disease (AD). This study reveals a substantial alteration in the monomer/oligomer distribution of Aβ_1-40,_ concomitant with re-direction of fibril formation, induced by the natural product interaction. These alterations on the Aβ_1-40_ aggregation pathway are most prominent for trans-crocin-4 (TC4). Use of ESI-IMS-MS, electron microscopy alongside Thioflavin-T kinetics, and the interpretation of 3-dimensional Driftscope plots indicate a correlation of these monomer/oligomer distribution changes with alterations to Aβ_1-40_ amyloid formation. The latter could prove instrumental in the development of novel aggregation inhibitors for the prevention, or treatment of AD.

## Introduction

Abnormal protein and peptide aggregation represent the key event in numerous chronic neurodegenerative diseases such as Alzheimer’s Disease (AD) and other central nervous system diseases such as Parkinson’s Disease (PD) and Huntington’s Disease (HD). In our ageing society, AD is the most common cause of senile dementia and it is associated by impaired synaptic function, a reduction in brain cell mass, loss of cognitive ability and premature death^1^. AD has a major impact on human health along with immense societal impact and important economic consequences. As the life expectancy increases, it is projected that the number of people > 65 years with AD in the United States will at least triple to 15 million by 2050 from 5 million currently affected^2^. There is, therefore, an urgent need to find a means to halt and even reverse this trend.

The molecular mechanisms underlying the pathogenesis of AD have not yet been elucidated. Nevertheless, it is widely believed that AD is associated with abnormal accumulation of misfolded peptides, which aggregate and cause damage to neurological tissues. The two characteristic histopathological lesions found in patients with AD are extracellular deposits of beta-amyloid peptide (Aβ) forming senile plaques (SP) and intraneuronal neurofibrillary tangles (NFTs) composed of abnormal filaments of hyperphosphorylated tau protein^3^. To date, AD can only be confirmed post mortem, and the Aβ peptide, present in the Aβ_1-40_ and Aβ_1-42_ forms, is the major proteinaceous component found in plaques. One of the most prevalent mechanisms of AD pathogenesis appear to be the *amyloid cascade hypothesis* of Aβ deposition^4^, whereas all other molecular and histopathological characteristics including intracellular NFT, neuronal loss and vascular damage, are downstream events of the Aβ deposition^5^. Even though the correlation between protein aggregation and nervous system degeneration remains largely elusive, these disease-specific aggregated peptides have apparent diagnostic and even therapeutic implications^6,7^. In particular, the role of Aβ in the neuropathology of AD is undisputed even though the causative link between Aβ and impaired neuronal function is still under investigation. Oxidative stress and neuroinflammation have also been identified among the principal pathways of neurodegeneration^8,9^, thus rendering antioxidants as one of the prime candidates for anti-aggregation compounds that may prevent aggregation/oligomerization of Aβ and/or promote clearance of Aβ aggregates. In particular, inhibiting formation of Aβ oligomeric states and amyloid fibril by small molecule binding to the peptide has been proposed as a viable therapeutic strategy^10^. Both the Aβ_1-40_ and Aβ_1-42_ variants have been shown to exist as monomeric forms in rapid equilibrium with the corresponding soluble oligomers, and they self-assemble via different and distinct pathways as previously reported^11,12^. Here we evaluate the anti-amyloid activity of dietary antioxidants and especially isolated components from the stigmas of saffron (*Crocus sativus* L.) by electrospray ionization-ion mobility spectrometry- mass spectrometry (ESI-IMS-MS). ESI-IMS-MS is an emerging tool for structural characterization whereby complex mixtures of species in solution can be resolved on the basis of their shape and/or charge without prior separation and each component mass measured within the same experiment^13^. ESI-IMS-MS has been previously employed to provide insights into the oligomerization of Aβ_1-40_ and Aβ_1-42_ peptides^14,15^. In addition, ESI-IMS-MS has shown to be a suitable method to provide insights into the interactions of small molecules with macromolecules^16^, as well as to elucidate the distinct species where the small molecule binds^17–19^. It is a rapid in vitro screening method providing stoichiometry and conformer information requiring a small amount of sample without any labeling^20^. In this study, the ESI MS screening experiments were carried out with the most abundant Aβ_1-40_ peptide variant. In case of the Aβ_1-42_ variant, the observed ESI mass spectral signal was rapidly diminished over time presumably due to its aggregation and the subsequent clogging of the electrospray tip. Therefore, the preliminary experiments with the Aβ_1-42_ peptide were very difficult to control in the instrumentation employed in this study (QqTOF and Synapt G2 HDMS systems).

**Figure 1:**
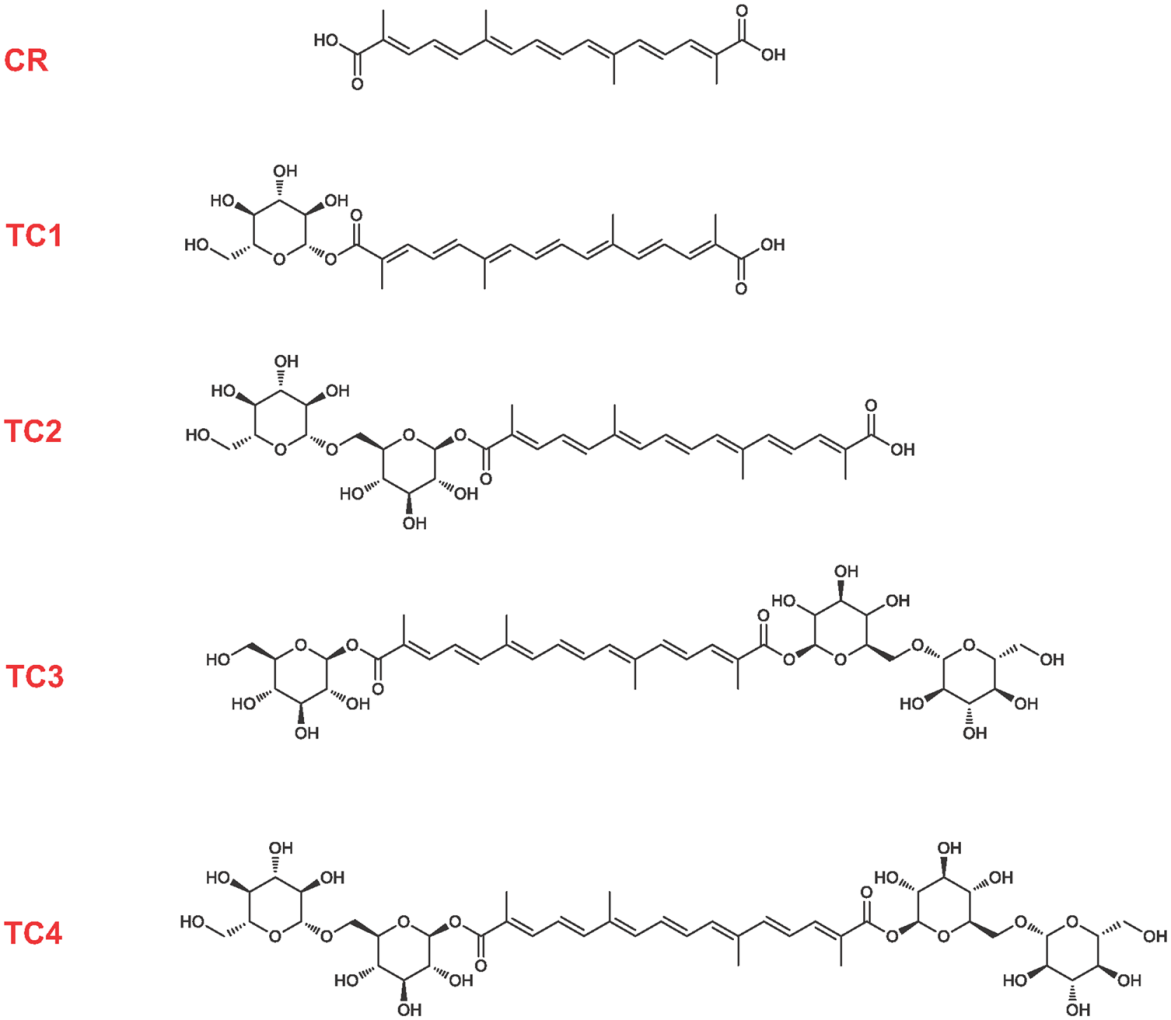
Chemical structures of selected crocin ester glycosides isolated from the stigmas of *Crocus sativus* L. The crocins are divided according to the number and the position of the β-l-glucopyranosides attached to the central carotenoid unit (Crocetin- CR), comprising crocetin mono- and bis-ester glycoside compounds with the end-substituents being mono- (Glycosyl- TC1) and bi- (1β-d-gentiobiosyl- TC2, TC3 and TC4) disaccharide moieties.

Several studies have indicated the neuroprotective effects of several bioactive compounds usually contained in extracts (e.g., berries, red-wine polyphenols) by modulating β-amyloid-dependent and independent mechanisms^21,22^. There are numerous reports for the health-promoting properties of saffron such as antidepressant^23^, anti-inflammatory^24^, anticancer^25^, and antiatherogenic effects^26^. Nevertheless, there is limited information on neuroprotection by plant-derived and dietary antioxidants such as those derived from *Crocus sativus* L. In general, crocins are glycosyl esters of crocetin, a water-soluble carotenoid dicarboxylic acid, which mainly occurs in all-*trans* form (even though the *cis*-isomers have also been identified). Likewise, crocins could be divided according to the number and the position of the β-l-glucopyranosides attached to the central carotenoid unit, comprising crocetin mono- and bis-ester glycoside compounds with the end-substituents being mono- (Glycosyl- TC1), bi- (Gentiobiosyl- TC2, TC3 and TC4) and tri-saccharide (Gentiotriosyl and Neapolitanosyl) moieties^27^(Fig. 1). This study demonstrates the successful screening of isolated and well-characterized components of *Crocus sativus* L. in terms of their binding to Aβ_1-40_ and the subsequent alteration in pre-fibrillar Aβ_1-40_ species populated in the presence of these compounds. The morphologies of the resulting Aβ_1-40_ aggregates in the absence and presence of the natural product (NP) were assessed by transmission electron microscopy (TEM). The choice of the Aβ_1-40_ was essential because we did not want fibril formation to take place too rapidly in order to increase chances to observe the NP effect within the time period in the ESI-IMS-MS and TEM-aggregation studies. The goal of our study was to observe the interactions of monomer and oligomers with the NP, the alteration in the distribution of monomer and oligomer conformers induced by the NP and the correlation of these changes with potential inhibition of Aβ_1-40_ amyloid formation. The latter may be significant for identifying targets and designing putative inhibitors, and it could be invaluable towards shedding some light into the pathways of oligomer formation in amyloid disease.

## Methods

### Sample preparation for MS

**Figure 2:**
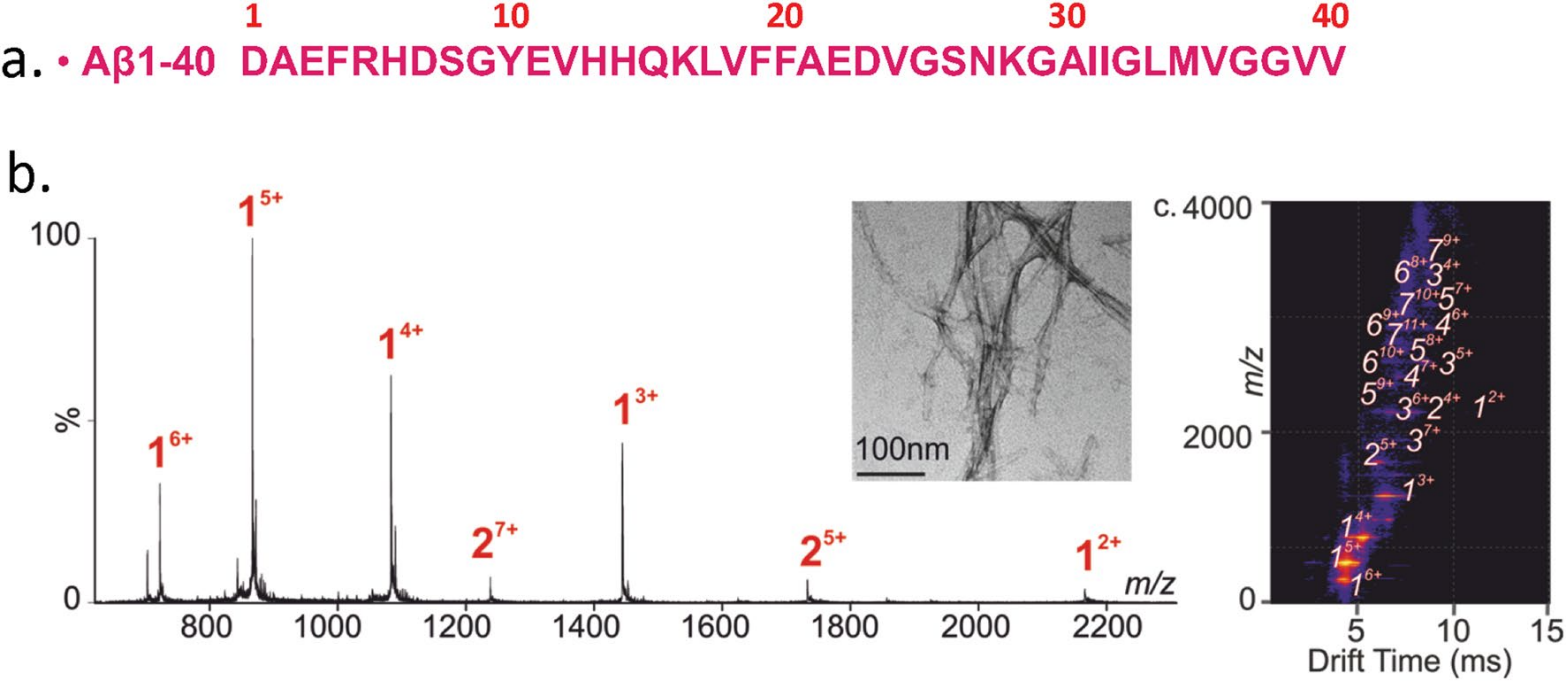
Aβ_1-40_ forms an array of oligomeric species during fibril formation. (**a**) Primary sequence of the Aβ_1-40_ peptide. (**b**) The ESI mass spectrum of the Aβ_1-40_ peptide acquired on a Synapt HDMS mass spectrometer. The oligomer order is denoted above the mass spectral peak along with the positive-charge state of the ion as superscript. (**c**) The ESI IM-MS Driftscope plot of the Aβ_1-40_ monomer (1) through the heptamer (7). The TEM image of the Aβ_1-40_ sample after 48 h of incubation (water pH 5.8, quiescent, 25 °C) is shown in the inset (scale bar 100 nm).

Aβ_1-40_ (M_r_ 4329.9) peptide (Fig. 2a) was purchased from Bachem GmbH (Catalog number H-1194.1000, Lot number 1075609; Weil am Rhein, Germany). Lyophilized Aβ_1-40_ was resolubilized in DMSO at a peptide concentration of 3.2 mM and diluted into water (pH 5.8), 1% (v/v) DMSO at a final peptide concentration of 32 μM. The pH ~ 6.0 was essential for maintaining the noncovalent interactions between the Aβ peptide and the *Crocus*-isolated compounds. The sample was centrifuged at 13,000* g* (4 °C, 10 min) prior to MS analysis to remove any insoluble aggregates that may have formed. All samples were incubated at 25 °C in 96-well plates without agitation. An aliquot of this freshly prepared Aβ_1-40_ solution in water (pH 5.8) was mixed with a tenfold molar excess (320 µΜ) aqueous solution of the ligand, which was an isolated component from the stigmas of saffron (*Crocus sativus* L.). Major biologically active components from the stigmas of *Crocus sativus* L. were extracted, separated and isolated from dried stigmas of saffron flowers provided by Cooperative De Safran Krokos (Kozani, Greece) by semi-preparative HPLC and structurally characterized as previously described^27^. The isolated components from *Crocus sativus* L. listed in Fig. 1 were screened for antiamyloidogenic activity.

### Enzymatic digestion for mapping analysis

Tryptic enzymatic mapping was used to study the interaction site between Aβ peptide and TC4. The Aβ peptide and its mixtures with the ligand were dissolved in 25 mM ammonium bicarbonate (Merck) and the pH of the solution was adjusted to 8.4 with 10% aqueous ammonium hydroxide solution. Then TPCK-treated trypsin (Worthington Biochemical Co., Lakewood, NJ, USA) was added at an enzyme:substrate ratio of 1:50 (w/w). The enzymatic reaction was carried out at 37 °C and quenched after 14 h by adding 2.5% acetic acid and deep-freezing of the samples using the procedure previously described^28^.

### ESI-MS and ESI-IMS-MS analysis

Accurate mass measurements of the crocus-derived bioactive components (10,000 FWHM resolution using the leucine-enkephaline standard as a lock mass) and preliminary analysis of the Aβ_1-40_-crocin complexes was performed on-line on a Waters Premier quadrupole reflectron time-of-flight (QqTOF) high-resolution instrument (Waters Corp., Manchester, UK) equipped with an ESI source in the positive ion mode. ESI MS mapping analysis of the Aβ_1-40_:TC4 complex trypsin-generated fragments was carried out on the QqTOF instrument following the procedure previously described^28^. IMS-MS analysis was performed on a Synapt G2 HDMS quadrupole-time-of-flight mass spectrometer (Waters Corp., Manchester, UK), equipped with a Triversa NanoMate (Advion Biosciences, Ithaca, NY, USA) automated nano-ESI interface. The instrument, described in detail elsewhere^29^, has a traveling-wave IMS device situated between the quadrupole and the time-of-flight analysers.

Aβ_1-40_ and crocetin samples were analysed using positive mode nanoESI with a capillary voltage of 1.7 kV and a nitrogen nebulising gas pressure of 0.8 psi. The following instrumental parameters were used, as described previously^19^: cone voltage 30 V; source temperature 60 °C; backing pressure 1.6 mBar; ramped travelling wave height 7–20 V; travelling wave speed 300 m/s; IMS nitrogen gas flow 20 mL/min; IMS cell pressure 0.55 mBar. Data were acquired over the range m/z 100–6,000. Data were processed by use of MassLynx v4.1 and Driftscope software supplied with the mass spectrometer^18^. Mass calibration was achieved using caesium iodide solution, prepared by dissolving the compound in 50% (v/v) water/ isopropanol to a concentration of 2 mg/mL.

### Transmission electron microscopy (TEM)

In the preliminary analyses—carried out at U. Leeds-, the TEM images of each peptide solution were acquired after 48 h of incubation in water (pH 5.8) at 25 °C in low binding tubes (MAXYMum Recovery tubes, Axygen), using a JEM-1400 (JEOL Ltd., Tokyo, Japan) transmission electron microscope. Carbon grids were prepared by irradiation under UV light for 30 min and stained with 4% (w/v) uranyl acetate solution as described previously^30^.

In the time-series TEM experiments—carried out at KTH/Karolinska Institutet-, Aβ_1-40_ peptide and the ligand TC4, both in 1% (v/v) DMSO, were mixed in 1:6 molar ratio at a final peptide concentration of 2 µM. Aβ_1-40_ and Aβ_1-40_:TC4 samples were made at time points 0 (start), 1 h, 4 h and 24 h. Incubation was performed at +4 °C in order to slow down the aggregation and the fibril formation. However, for the last 3 h (21–24 h) the samples were transferred to room temperature (+21 °C) in order to help the process progress as much as possible for the last imaging time point (24 h). 3 µl of the sample was applied onto glow-discharged 400 mesh Cu-grids, containing a continuous carbon support film. After 1 min incubation excess sample was blotted away, the grid washed in two drops of water and stained with 2% (w/v) uranyl acetate for 30 s. After blotting the stain with filter paper, grids were air-dried. Images were collected with a JEOL JEM2100F (JEOL Ltd., Tokyo, Japan) transmission electron microscope operated at 200 kV using a Tvips TemCam-F415 CCD detector.

### Thioflavin T (ThT) fluorescence assays

Samples were added to a 96-well plate (Corning Costar 3915, Corning Life Sciences, Amsterdam, The Netherlands), sealed with clear sealing film and incubated in a FLUOstar OPTIMA plate reader (BMG Labtech, Aylesbury, Bucks, UK) for 5 days at 25 °C without agitation. Each 100 μL sample contained ThT (100 μM) and peptide (32 μM) in water (pH 5.8), in the presence or absence of 320 μM isolated NP, with a 1% (*v/v*) final concentration of DMSO, as described previously^20^. The thioflavin-T studies used excitation and emission filters of 430 and 485 nm, respectively.

## Results and discussion

### Formation of Aβ oligomeric assemblies and fibrils

Consistent with previous results^13,14,20^, the ESI mass spectrum of Aβ_1-40_ shows primarily multiply charged ions of the monomer, ranging from +2 to +6 (Fig. 2b). Furthermore, weak signals corresponding to multiply charged ions of the dimer (+5 to +7) were also observed in the ESI mass spectrum. These ions were more pronounced in the analysis of Aβ_1-40_ by ESI-IMS-MS, where signals corresponding to Aβ_1-40_ oligomers ranging from dimer to heptamers were readily detected in the ESI-IMS-MS driftscope plot of the Aβ_1-40_ monomer (Fig. 2c), thus demonstrating formation of Aβ_1-40_ aggregated species. This is apparently due to the formation of Aβ_1-40_ oligomers (dimers to heptamers) early in the process of fibril assembly. Co-populated oligomeric ions with the same m/z were separated and identified individually by ESI-IMS-MS (e.g., monomer^2+^, dimer^4+^ and trimer^6+^). Additionally, multiple charge states, ranging from +2 to +8, were observed for the Aβ_1-40_ oligomers. The absence of the higher-order oligomers (> 7-mer) indicates that they are either too lowly populated to be observed under these conditions or are not significantly occupied under these conditions. The morphologies of the resulting Aβ_1-40_ aggregates were assessed by TEM, which showed the formation of long straight fibrils after five days at 25 °C in water (pH 5.8) (Fig. 2b, inset). Time course analyses suggest that the oligomers observed using ESI-IMS-MS are incorporated into fibrils as aggregation proceeds.

### Modulation of Aβ aggregation by natural products

There may be several possible mechanisms of intervening in the process of Aβ aggregation, including inhibition of oligomer formation, or fibril formation, or both. In this study, we assessed the ability of isolated and well-characterized components of *Crocus sativus* L. to bind to monomeric Aβ_1-40,_ and inhibit the formation of Aβ_1-40_ oligomers, by ESI-IMS-MS. In addition to mass spectrometric analyses, fibril formation was monitored using thioflavin T (ThT) fluorescence and the morphologies of the resulting aggregates were assessed using negative stain TEM. The formation of 1:1 noncovalent complex of Aβ with certain antioxidants such as oleuropein (OE) and melatonin (M) has been previously demonstrated by ESI-MS^31,32^, and the interaction of these compounds with the hydrophobic region of the peptide (peptide region 17–28) has been reported by NMR^33^ and ESI-MS proteolytic mapping studies^28^. The noncovalent interaction of the *Crocus sativus* L.-derived components with Aβ_1-40_ was first assessed using ESI-MS.

**Figure 3:**
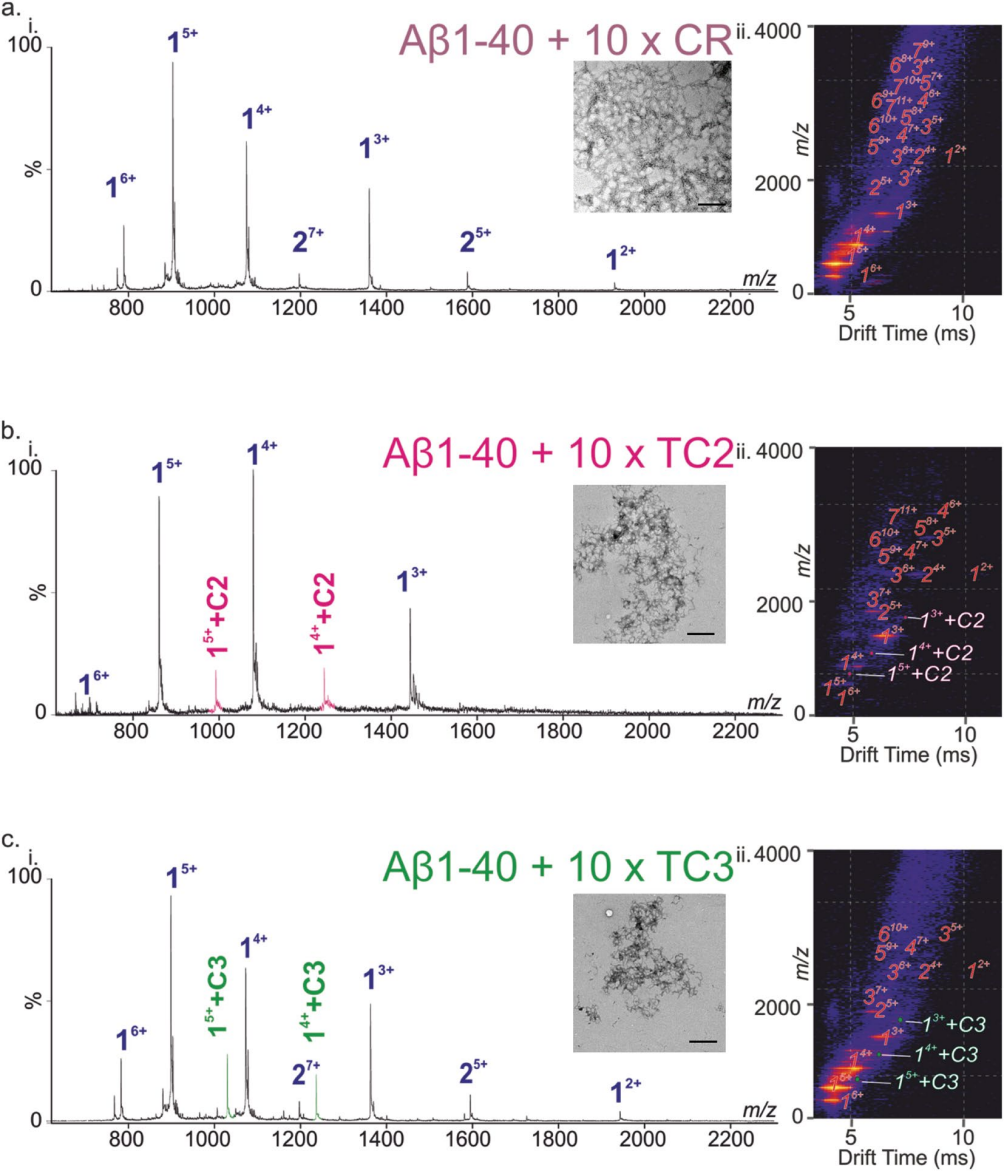
Inhibition screening of the Aβ_1-40_ amyloid assembly by CR, TC2 and TC3. The ESI mass spectra of the Aβ_1-40_ peptide in the presence of a tenfold molar excess of (**a-i**) CR, (**b-i**) TC2 and (**c-i**) TC3. The oligomer order is denoted above the mass spectral peak along with the positive-charge state of the ion as superscript. ESI IM-MS Driftscope plots of the Aβ_1-40_ peptide oligomers in the presence of a tenfold molar excess of (**a-ii**) CR, (**b-ii**) TC2 and (**c-ii**) TC3, showing monomer (1) through the heptamer (7) or hexamer (6) in the case of TC3. The respective TEM images of the samples after 48 h of incubation (water pH 5.8, quiescent, 25 °C) are shown in the insets. Scale bars 500 nm.

**Figure 4:**
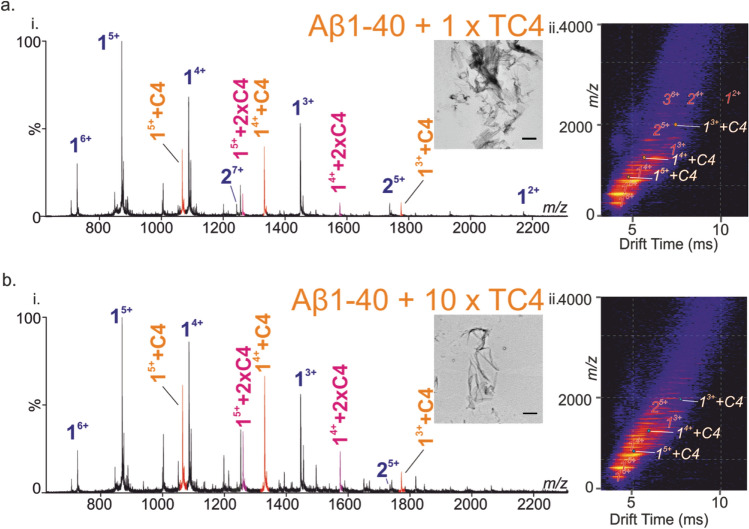
Inhibition of the Aβ_1-40_ amyloid assembly by TC4. The ESI mass spectra of the Aβ_1-40_ peptide in the presence of equimolar (**a-i**) and a tenfold molar excess (**b-i**) of TC4. ESI IM-MS Driftscope plots of the Aβ_1-40_ peptide in the presence of equimolar (**a-ii**) and a tenfold molar excess (**b-ii**) of TC4 showing monomer (1) through the trimer (3) or dimer (2) in the case of a tenfold excess of TC4. The TEM images of the samples after 48 h of incubation (water pH 5.8, quiescent, 25 °C) are shown in the insets. Scale bars 2 μm.

ESI-MS analysis of the Aβ_1-40_:CR solution yielded no signals corresponding to the formation of a noncovalent complex (Fig. 3a-i). On the contrary, ESI-MS screening of the other *Crocus sativus* L.-isolated components (Fig. 1), trans-crocin-2 (TC2), trans-crocin-3 (TC3) (Fig. 3b-i, c-i), and trans-crocin-4 (TC4) (Fig. 4), revealed noncovalent interactions with Aβ_1-40_. The specific nature of these interactions was evaluated at lower concentrations and different molar ratios of Aβ_1-40_:crocin. The ESI signals corresponding to the noncovalent complex of Aβ_1-40_ with crocins were present at all concentrations (data not shown), thus suggesting the specificity of these interactions. The ESI mass spectra of the Aβ_1-40_:crocin samples exhibited two multiply charged ion envelopes corresponding to the +2, +3, +4, +5 and +6 charge states of Aβ_1-40_ and the +4 and +5 charge states of the 1:1 Aβ_1-40_:NP noncovalent complex, respectively. The signals of the Aβ_1-40_:NP noncovalent complexes are more pronounced as the size of the sugar component is increased (TC4 > TC3 > TC2), and this is clearly shown in the ESI-MS analysis of the Aβ_1-40_:TC2, Aβ_1-40_:TC3 and Aβ_1-40_:TC4 samples. This is revealed by the intensity of the +4 and +5 charge state signals corresponding to the 1:1 Aβ_1-40_:Crocin noncovalent complexes, which is increasing in the following order TC4 > TC3 > TC2 (Figs. 3b-i, c-i, Fig. 4a-i, b-i). In case of the Aβ_1-40_:TC4 sample, deconvolution of the observed ion envelopes gave rise to M_r_ of 4329.8 and 5306.8, with the latter being in good agreement with the theoretical average mass of 5306.3 for the 1:1 Aβ_1-40_:TC4 noncovalent complex. Moreover, the observed signals corresponding to the noncovalent association were more pronounced when the molar ratio was increased to 1:10, where incorporation of a second TC4 ligand was observed as shown in Fig. 4b-i. It should be noted that ESI-MS tryptic mapping studies of the Aβ_1-40_:TC4 noncovalent complex showed that TC4 interacts with the tryptic peptide 17–28, as evidenced by the doubly charged signal at m/z 1151.5038. This agrees with the previously reported results with OE binding to the hydrophobic region of the peptide (residues 17–28)^28^.

Following the ESI-MS analyses, ESI-IMS-MS was used to evaluate the crocin interaction with the Aβ_1-40_ monomer and several of its oligomers. In case of CR, the binding was not detectable (or very weak) and Aβ_1-40_ formed aggregates (dimer through heptamers) in a fashion comparable to when incubated alone (Fig. 3a-ii).

Similar fibril formation compared to Aβ_1-40_ alone was observed by TEM for both 1:1 and 1:10 molar ratio of CR after 48 h of incubation (Fig. 3a-i, inset). When Aβ_1-40_ was incubated in the presence of TC2, a small amount of binding was observed using ESI-IMS-MS especially at the Aβ_1-40_:NP 1:10 molar ratio (Fig. 3b-ii). Formation of Aβ_1-40_ aggregates was observed using TEM, even though the intensity of the higher order oligomers was slightly decreased when TC2 was in 1:10 excess. Employing the same ESI-IMS-MS screening approach, binding of TC3 to Aβ_1-40_ was observed at both 1:1 and 1:10 molar ratio. Formation of Aβ_1-40_ aggregates from dimer through hexamer was observed with slightly decreased higher order oligomer intensity especially in the case of 1:10 Aβ_1-40_:TC3 (Fig. 3c-ii). That was accompanied by the observation of significantly fewer, short protofibrils by TEM after 48 h (Fig. 3c-i, inset). It should be noted that TEM images of Aβ_1-40_ incubated with TC3 for two weeks showed longer fibrils, with different morphology to those observed for Aβ_1-40_ alone, suggesting that this interaction is slowing, but not abolishing, fibrillation of Aβ_1-40_.

In case of the bis-ester glycoside containing crocin (trans-crocin-4; TC4) component, the peak corresponding to bound Aβ_1-40_ monomer was observed at high intensity at both 1:1 and 1:10 Aβ_1-40_:TC4 molar ratio (Fig. 4a, b). Further, oligomerization of Aβ_1-40_ is significantly diminished at both 1 × and 10 × excess of TC4. The most significant characteristic of TC4 binding to Aβ_1-40_ is the apparent re-directing of amyloid fibril formation as observed by TEM (Fig. 4a, b, insets). Large ribbon-like structures are observed instead. The stark difference in morphology of the aggregates formed in the presence and absence of TC4 suggests that this small molecule re-directs the aggregation pathway from forming typical amyloid fibrils to aggregates of a different structure. The re-directed pathway does not involve significant population of low-order oligomeric states that are visible using ESI-IMS-MS.

**Figure 5:**
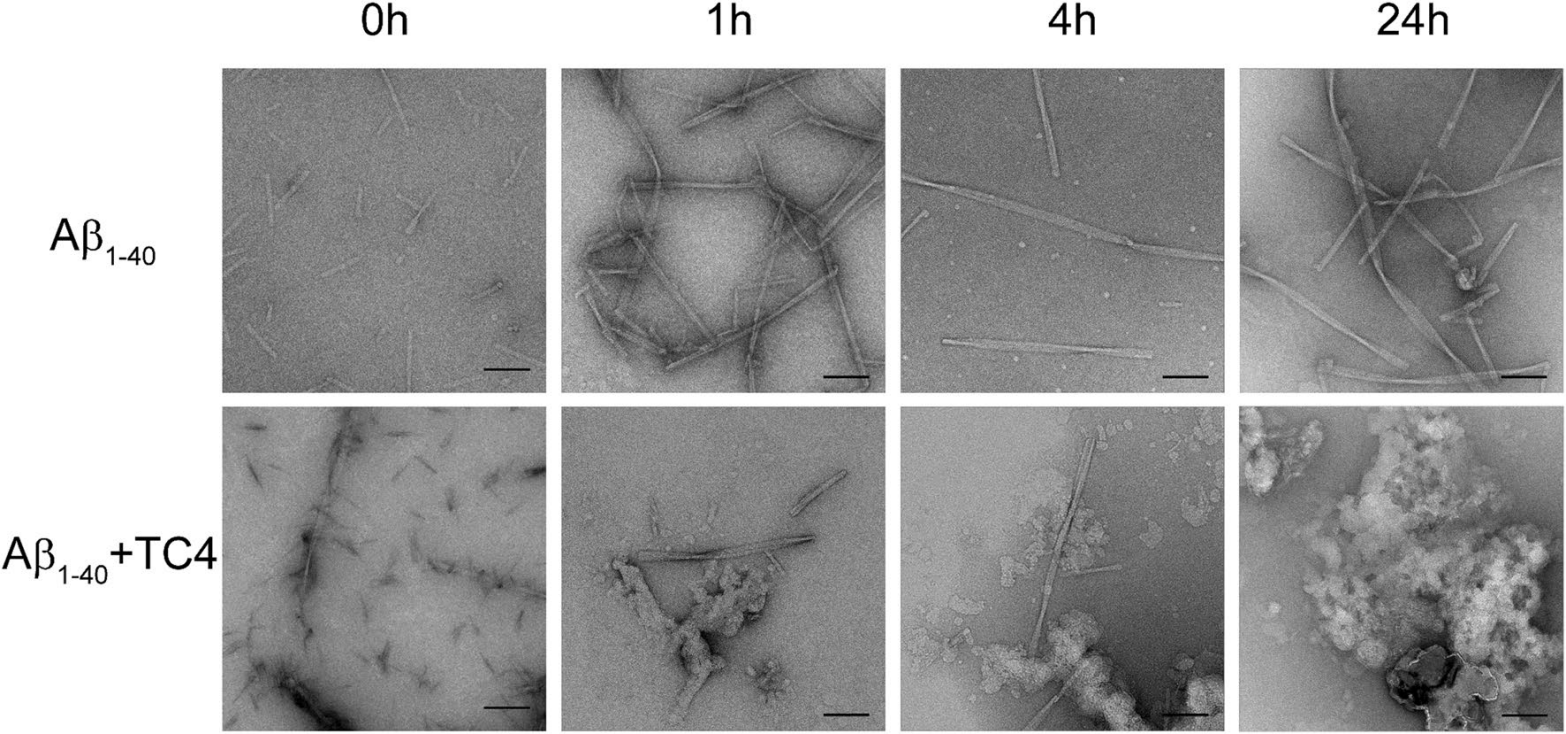
A time-series of Aβ1-40 peptide sample without/with TC4 analyzed by negative stain TEM. Samples were prepared at start of the experiment and after 1, 4 and 24 h incubations. Scale bars 100 nm.

In a more detailed time-series TEM study, Aβ_1-40_:TC4 (molar ratio 1:6) samples were prepared at time points 0 (start), 1 h, 4 h and 24 h. Incubation was done at +4 °C, and then at room temperature (+21 °C) for the last 3 h (21–24 h). During the time series the Aβ_1-40_ sample showed maturation towards long, broad and twisted fibers, whereas in the presence of TC4 this led to obvious aggregates in the vicinity of the fibers. At later time points these aggregates had grown larger and at 24 h single fibers were rarely detected (Fig. 5).

### Insights into the inhibition mechanism of Aβ aggregation

The ESI-IMS-MS approach employed in this study has demonstrated the significant decrease in the Aβ_1-40_ oligomerization in the case of certain crocin components, such as TC4, which bind significantly to the peptide. Nevertheless, the mechanism by which these small molecules inhibit amyloid formation is largely unknown. In this study, analysis of ESI-IMS-MS Driftscope plots reveals that the formation of Aβ_1-40_ aggregates, i.e., dimer through heptamer, was mainly unaffected by the presence of CR at both 1:1 and 1:10 Aβ_1-40_:CR molar ratio (Fig. 3a-i). When a small amount of binding with the ligand was observed (TC2 and TC3) the intensity of the signal arising from higher order oligomers was decreased especially in the case of 10 × excess of the crocin component.

**Figure 6:**
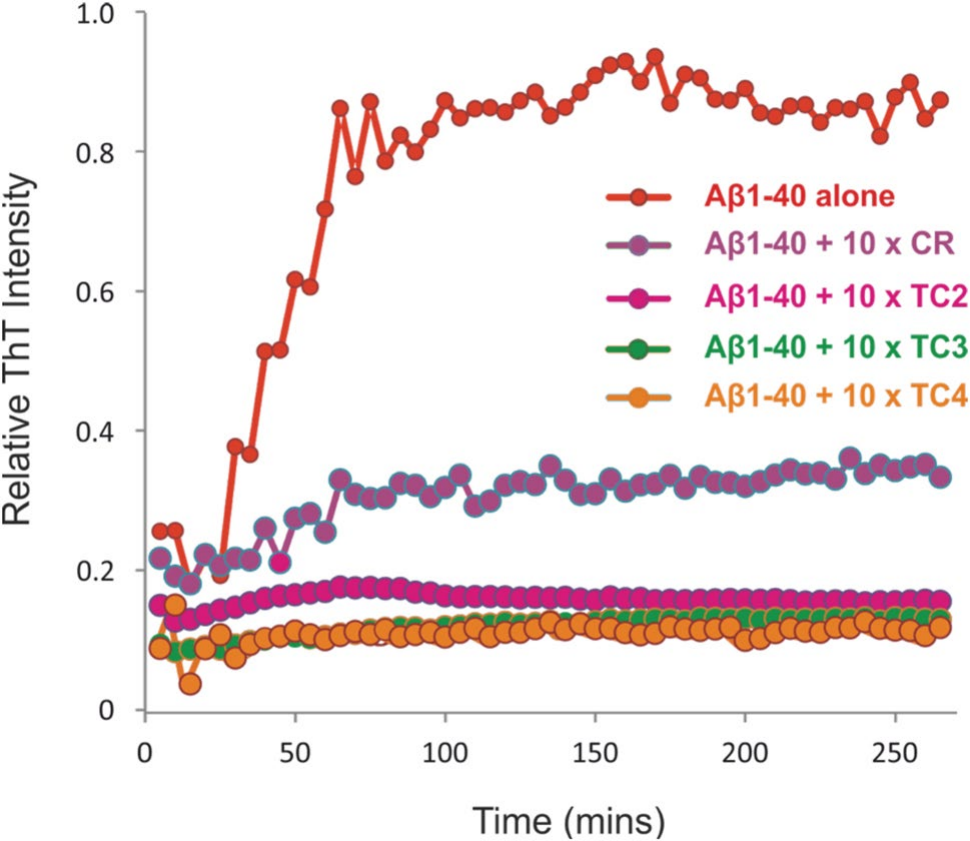
Inhibition of the Aβ amyloid assembly by Crocins. ThT fluorescence intensity over time of Aβ peptide alone (red circles) (32 μM peptide in water, pH 5.8, 25 °C, quiescent) and in the presence of the isolated crocin components containing 0–4 carbohydrate moieties, i.e., CR (purple), TC2 (cyan), TC3 (green) and TC4 (orange) at 10:1 molar ratio.

In case of TC4 there is significant binding to monomeric Aβ_1-40_, which results in substantial alteration in the monomer/oligomer distribution induced by the crocin interaction. It seems that TC4 binding to the monomeric peptide causes restrain of self-assembly resulting in lack of oligomers, as shown by the significant decrease in the intensity of the higher order aggregates (trimer and higher) (Fig. 4a-ii). This is more pronounced in case of 1:10 Aβ_1-40_:TC4 molar ratio, where there is a complete absence of all aggregates in the ESI-IMS-MS Driftscope plot (Fig. 4b-ii), thus enforcing the notion of a dose-dependent prevention of oligomerization. The fibril formation was monitored using ThT fluorescence which showed a decrease in the formation of ThT-positive Aβ species on the addition of the crocin components (Fig. 6). In the absence of crocin components, Aβ_1-40_ forms ThT binding aggregates after a lag phase of ~ 1 h, whereas ThT signals were diminished in the presence of the crocin compounds. The addition of the crocin components confirmed the partial or full inhibition of amyloid formation. In particular, the formation of ThT-positive fibrils in vitro was partially reduced in the presence of CR, whereas it was more suppressed with the addition of the TC3 (green) and TC4 (orange) components. The latter alters the distribution of charge states and the monomer/oligomer distribution as shown in Fig. 4b-ii. This significant inhibition of the formation of ThT-positive species observed only in the presence of excess TC4 (Fig. 6) and the apparent absence of typical long, straight amyloid fibrils material as observed by TEM, suggest that binding of TC4 may redirect Aβ_1-40_ peptide onto an alternative pathway that results in long ribbon-like structures^34^. It is important to note that these ribbon-like assemblies have been reported to have cross-*β* structure^35–37^.

The occurrence of Aβ_1-40_ aggregates with altered morphology is also shown in the more detailed time-series TEM study of the Aβ_1-40_:TC4 samples, where normal fiber maturation and growth tries to proceed but is counteracted by the effect of TC4 on Aβ fibers (Fig. 5). It is possible that some aggregates and small thread-like structures, most likely TC4, are attached to Aβ. This could lead to disaggregation of normal fibrillar structures and aggregation of the material in large aggregates. Additionally, the binding of more than one TC4 ligands could promote the stabilization of the monomeric charged states of Aβ_1-40_. That was observed for all the observed charge states (3+, 4+ and 5+) of the monomer, where one and two TC4 ligands are bound when Aβ_1-40_ is incubated with 1:1 molar ratio of TC4. The binding of two ligands is more pronounced when TC4 is added in a tenfold excess over the Aβ_1-40_ peptide. Furthermore, the presence of the TC4 ligand causes an apparent alteration in the relative abundance of the monomeric ions of Aβ_1-40_, especially the 3+ and 4+ charge states, which are more populated when the TC4 ligand is bound to Aβ_1-40_, as it was observed in the case of hIAPP inhibition by EGCG^19^. Therefore, it seems that direct binding of TC4 to the monomeric peptide does initiate conformational changes, which in turn alter the charge state distribution pattern, i.e., enhancing the 3+ and 4+ ions compared to the 5+ charged state of the monomer.

## Conclusions

This study demonstrates that dietary antioxidants and especially isolated components from the stigmas of saffron (*Crocus sativus* L.) may be effective in re-directing the pathway of Aβ aggregation. This ESI-IMS-MS approach comprises a powerful tool for detecting noncovalent interactions of biomolecules with NPs using small amounts of protein and ligand, as well as for providing information on the alteration in the monomer/oligomer distribution induced by the NP. The presented ESI-IMS-MS data combined with the TEM study of the Aβ_1-40_:crocin samples reveal a substantial perturbation of the typical amyloid fibril forming pathway and alteration in the monomer/oligomer distribution of Aβ_1-40_ induced by the crocin interaction, especially in the case of TC4. The use of ESI-IMS-MS and the interpretation of the 3D Driftscope plots indicate an apparent correlation of these monomer/oligomer distribution changes with the re-directing of Aβ_1-40_ amyloid formation. It is possible that TC4 binding to Aβ monomers and dimers and the ensuing redirection of amyloid fibril formation may also shift the equilibrium from smaller and more toxic oligomers towards stable and organized fibrils, as previously suggested^38^. These results underline the utility of ESI-IMS-MS as a powerful screening tool in this area of neuroscience for identifying lead compounds, which could act as protective or even therapeutic agents against AD. In addition, this study shows the significant role that these plant-derived compounds, such as TC4, can play in their actual form (or as derivatives), and their potential exploitation in the form of nutraceuticals towards the prevention and/or treatment of AD. This is in good agreement with the recent demonstration of TC4 for suppressing key molecular pathways of AD pathogenesis^39^. In that study, it was also observed that TC4 did not compromise cell viability of neuron-like cells at biologically relevant range of concentrations and incubation times, and it was even enhancing cell growth at the highest tested concentrations^39^, thus rendering it a promising tool in the prevention and potentially the treatment of AD. Finally, the potential use of nutraceuticals will also have a positive impact to preserve and enhance the environment and natural resources, and it will provide a stimulus for extensive cultivation of some of these plants in the originating countries.
