# Publisher Correction: Crocus-derived compounds alter the aggregation pathway of Alzheimer’s Disease - associated beta amyloid protein

**DOI:** 10.1038/s41598-021-82907-9

**Published:** 2021-02-02

**Authors:** Nikolaos Stavros Koulakiotis, Pasi Purhonen, Evangelos Gikas, Hans Hebert, Anthony Tsarbopoulos

**Affiliations:** 1 GAIA Research Center, Bioanalytical Department, The Goulandris Natural History Museum, 14562 Kifissia, Greece; 2 School of Engineering Sciences in Chemistry, Biotechnology and Health, Department of Biomedical Engineering and Health Systems, KTH Royal Institute of Technology, S‑141 52 Huddinge, Sweden; 3 Department of Pharmacy, National and Kapodistrian University of Athens, 15771 Athens, Greece; 4 Department of Pharmacology, National and Kapodistrian University of Athens Medical School, 75 Mikras Asias Street, 11527 Athens, Greece

Correction to: *Scientific Reports* https://doi.org/10.1038/s41598-020-74770-x, published online 23 October 2020

The original version of this Article contained an error in the title of the paper, where the phrase “Disease - associated” was incorrectly given as “Disease: associated”. This has now been corrected in the PDF and HTML versions of the Article.

